# Lister's relationship with patients: ‘A successful case’

**DOI:** 10.1098/rsnr.2013.0037

**Published:** 2013-05-29

**Authors:** Mary Wilson Carpenter

**Affiliations:** Queen's University, Kingston, Ontario K7L 3N6, Canada

**Keywords:** Joseph Lister, Margaret Mathewson, hospital patient rights, Edinburgh Royal Infirmary, tuberculosis, antisepsis

## Abstract

An important aspect of Joseph Lister's work that has received relatively little attention is his relationship with patients. However, a manuscript written by one of his patients, Margaret Mathewson's ‘A Sketch of Eight Months a patient, in the Royal Infirmary of Edinburgh, A.D. 1877’, provides detail about the surgeon as seen ‘from below’—that is, by a charity patient. Although excerpts from Mathewson's ‘Sketch’ have previously been published, an earlier version of the ‘Sketch’ has only recently been identified as such. That earlier version represents Lister not only as actively concerned with patient education, but also as strongly supportive of patients' rights, encouraging ward patients to report maltreatment at the hands of the staff.

## Introduction

In the summer of 1877, Joseph Lister returned from London, where he had been involved in negotiations for his new position as Professor of Clinical Surgery at King's College Medical School, to spend his final months in Edinburgh. On his visit to the Royal Infirmary of Edinburgh to examine his patients there, he found Margaret Mathewson's shoulder wound had made much less progress in healing than he expected.^1^ The wound seemed to have been wrenched apart by the dresser, rather than gently and carefully put through a full range of motions. In Mathewson's later written account, he asked:

Well do you think Mr —— did it from cruelty, or to cause you pain? No Sir, I think Mr —— did it so as I should not have a stiff joint afterwards, Sir. How do you think so? I think so Sir, as Mr —— told me I would be able to pull him around the bay near our place in Shetland when he came there to spend his holiday yet some day perhaps Sir (a laugh.) very good proof, gentlemen, the patient understands the term ‘a stiff joint.’^2^

Mathewson, a 29-year-old woman from the island of Yell in Shetland who had undergone the excision of her tuberculous shoulder joint by Lister some months before, explains to ‘the Prof’, as she calls him in her narrative, why she believes the student dresser did not intend her any harm, although he had manipulated her shoulder so forcefully during dressings as to cause great pain and copious bleeding. Lister replies with approval, pointing out to the students that the patient understands the term ‘stiff joint’. What Lister did not know was that Mathewson had, in fact, believed that the dresser was deliberately causing her pain for his own pleasure, and that she had confronted him, telling him that if he didn't treat her more gently, she could have him dismissed. He had not, however, treated her any more gently thereafter, but Mathewson had, for her own reasons, decided not to report his behaviour to the House Surgeon at the time, Dr Roxburgh.

Mathewson returned to her home after her discharge from the hospital on 23 October. By that time she had been seen by Lister and had her wound dressed by him, not only while she was an inpatient but once after she had been moved to the Convalescent Home. She obtained a pass from the Matron there to return to the Infirmary on 5 September because she had heard that this would be the Professor's final visit before his move to London. She was moved back into the Infirmary later in September and was still there on the date (1 October) when he delivered his introductory address at King's College, although she apparently knew nothing of that.^3^ More than a year later she first wrote a narrative account of her experience as a patient of Lister's, which she titled ‘A Sketch of Eight Months a Patient in the Royal Infirmary of Edinburgh, A.D. 1877’.^4^ This ‘Sketch’ is apparently the only known personal account by a charity hospital patient of Lister's and therefore provides a unique view of the already famous surgeon ‘from below’—from the perspective of a ward patient. Her narrative is of particular interest to health professionals and students of medical humanities because it demonstrates how a nineteenth-century hospital patient worked out the meanings of her experience as she repeatedly narrated it in letters home while she was in the hospital and then in writing and rewriting the ‘Sketch’ itself over a period of nearly a year, from December 1878 to September 1879. Her writings exemplify Trisha Greenhalgh and Brian Hurwitz's comment in the introduction to their work, *Narrative-based medicine: dialogue and discourse in clinical practice*:

You could make an objective list of the actions you performed over the last week, but if it were simply a ‘factual’ account, it would not *mean* anything. But if you *told* us what you had done in the last week, not only would your story acquire meaning, but in telling it, both you and the narrator and we the listeners would be compelled to reflect on it in order to gain a greater understanding of what had gone on.^5^

Particularly in the light of the recent discovery of two more versions of Mathewson's narrative—an incomplete manuscript dated 15 December 1878 and a complete narrative dated 26 July 1879—her repeated narrations of her experience not only represent her own reflections of what it was like to be a hospital patient, but the inclusion and exclusion of specific details also provide clues to the complexity and richness of this experience for her.^6^ Mathewson's repeated narrations of what happened to her also make a significant contribution to Lister studies. Aside from one article by W. B. Howie and S. A. B. Black,^7^ based on Mathewson's ‘Sketch’, very little has been written on Lister's relationship with patients. *Lord Lister*, the reverent biography written by his nephew, Sir Rickman John Godlee (1917), attests that ‘Lister was almost worshipped by his patients, rich and poor’, and goes on to quote the all-too-often quoted poem by William Ernest Henley, ‘The Chief’. The hagiography by a former student and colleague, John Rudd Leeson, *Lister as I knew him* (1927), includes several references to Lister's characteristic behaviour with patients—which may be summed up as always kind and good—but never consistently focuses on this issue.^8^ Richard B. Fisher's more recent biography, *Joseph Lister 1827–1912* (1977),^9^ similarly does not take Lister's relationship with patients, either private or charity, as a focus.

Mathewson's ‘Sketch’ should therefore be of signal importance in the fields of both patient studies and Lister studies, yet it has never been published in its entirety. More than 100 years after its composition, Martin Goldman, a science producer for BBC Radio Scotland, put together a book, *Lister Ward* (published posthumously in 1987). This work included excerpts from Mathewson's ‘Sketch’ and some of her letters, along with poems and letters by Henley, who had been a private patient of Lister's in the Royal Infirmary of Edinburgh earlier in the 1870s, and various writings by other medical contemporaries of Lister.^10^ The excerpts in Goldman's book, although misleadingly designated there as held in the Glasgow University Library, were based on a manuscript then owned by John Graham of Lerwick, Shetland, but now held in the Shetland Archives. This copy has only six pages in Mathewson's own hand, the rest having been copied by a friend of hers, Laurence Williamson, and is dated by the author 27 September 1879.^11^ The photocopy of the ‘Sketch’, from which I have quoted above, is an earlier version dated by the author 8 August 1879.

What has not been known until now is that the manuscripts of the ‘Sketch’ differ extensively, not only because many stylistic changes have been made but because Mathewson, as she said in the preface to the 27 September 1879 ‘Sketch’, decided to leave out some ‘insignificant items’ and put in others ‘more interesting’.^12^ Much of what was left out in this last known version is, to Foucauldian scholars of today, in fact not only ‘more interesting’ but extremely significant. Far from being the silenced, passive object of the ‘clinical gaze’, as has been argued about nineteenth-century hospital patients not only by Michel Foucault but by Nicholas D. Jewson and other scholars, Mathewson represents herself as the willing student of Lister and his staff, and staff as actively teaching patients such matters as how to do their own dressings.^13^ Mathewson believed that Lister deliberately lectured in English in the presence of patients so that they could benefit as much as medical and nursing students from what he said. Mathewson's account—even more surprisingly—also suggests that Lister encouraged patients to report on any staff member whom they felt was mistreating them. Yet in the later version of her ‘Sketch’, she toned down or omitted entirely much of what is most radical about her representation of Lister as ‘Prof’ to his patients as well as his other students. I will explore possibilities raised by a letter only recently found in the Shetland Archives as to why she might have made this particular revision.

My research in the Shetland Archives has demonstrated not only that Mathewson came from a family of prolific letter-writers, but also that she herself was an energetic and gifted writer. She wrote an autobiography of which only a fragment remains, but that fragment demonstrates the same vivid representation of the people she encountered and what she learned from them evident in her ‘Sketch’.^14^ The four copies of the ‘Sketch’ extant show her reworkings of her narrative and the emergence of new meanings—and new questions—for her. In her narrative, she progresses from being an ‘interesting case’ to a ‘successful case’. Lister, ‘the Prof’, emerges as generally ‘kind and good’, as attested by his biographers, but also as seriously concerned with both patient education and patients' rights, something that has not previously been described. And although Mathewson certainly believes Lister is the ‘best’ surgeon at the Royal Infirmary, she takes a strong personal interest in other members of his staff as well. It can even be argued that she identifies increasingly with that staff, although as one always subordinate to those elite members of the medical hierarchy, the doctors.

In the first part of this essay I discuss what Mathewson reported she had learned as a patient. This material was, for the most part, omitted from her revised last version (dated 27 September 1879) of her ‘Sketch’ and therefore was not included in the excerpts printed in Goldman's *Lister Ward*.^15^ In this essay I discuss and compare sections from the last two versions of Mathewson's narrative in which she describes her encounter with what she calls a ‘cruel dresser’. (The earlier two manuscripts, as described in note 4, were discovered too recently to be included in this study.) Both versions illuminate new aspects of Lister's relationship with patients.

## ‘But I expected it would be in Latin’

Although Mathewson was admitted to the Infirmary on 23 February 1877, she had to wait a full month before her operation. She was told by ‘the Drs’ that while her shoulder abscess kept open, they couldn't operate, and that they seemed to expect it to ‘burst’ by itself.^16^ During that month of waiting, Mathewson began what might be called her education as a student-patient. She was a keen observer, and her ‘Sketch’ includes many descriptions of treatments endured by other patients during this period, as well as accounts of dialogues with other patients on religious matters (she was an ardent Wesleyan Methodist and continually tried to persuade others they were *not* suffering because God wanted to punish them in this life before consigning them to everlasting punishment). What especially interested Mathewson were Lister's more innovative operations, such as performing a skin graft for a patient with varicose veins, and a blood transfusion for another. In both cases the same medical student (a Mr. Peddie) volunteered to donate the skin and the blood, and Lister rewarded his heroic generosity by having him sent home in a cab.^17^ Mathewson describes a baby brought in with bronchitis and an ‘heir sheir lip’, and how the baby had ‘his lip sewed up and his throat cut, and the bronchial tubes scraped and cleaned!!’^18^ The baby did very well and was sent home in two months, ‘Cured’. Mathewson was allowed to walk around the ward freely (and even outside), and as she learned more from her observations she took the opportunity to explain to other patients just what was being done to them and why. She even provides a drawing of the famous carbolic spray along with a detailed explanation of how it worked, and reassurance that it was not as dangerous as it looked.

When she was finally called into the theatre to be the subject of Lister's lecture, she found herself learning a great deal about her own case. In the earlier version of the ‘Sketch’, she notes that there were about ‘40 gentlemen and all the lecture was in English so I had the benefit of it too (but I expected it would be in Latin).’^19^ The parenthetical clause, that she had expected Lister to lecture in Latin, like most of the material Mathewson supplies about Lister's interest in teaching patients, has been removed from the later version of the ‘Sketch’. But the phrase attests to Mathewson's belief that Lister was lecturing in English with the *specific* intention of allowing the patient to ‘benefit’ from what he was saying, a belief she elaborates further in the earlier version of the ‘Sketch’. Noting that, while she was seated in the operating theatre facing the students, the ‘Prof’ asked her almost the same questions as he had earlier, she then repeats his remarks to the students.

‘Thus Gentlemen this is a very singular case of Consumption of the lungs, both of which were a little effected but it had providentally taken a turn and gone off the lungs and seated in the shoulder joint there forming a circumfical abscess. Also here's another glandular abscess on or near the collar bone the patient must have been no stranger to suffering as you see here the form of the chest bones, also the singular shape of the shoulder joint.’^20^

As indicated by her exact repetition of Lister's words (this is the second time she has repeated his diagnosis in the ‘Sketch’), this patient understood the import of what he was saying. She interjects in her account, ‘I was very much excited by this time so much so that I felt the cold perspiration running down my forehead!’^21^ This, she comments, the Prof observed and so, patting her on the arm, told her to ‘now turn your back on these gentlemen’, a move that faced her in the same direction as the listening students.

but on turning round my feelings and fears were more aroused by looking first on ‘The Black Board’, and there seeing the diagram of my arm chalked on it in its then swelled state, also the natural shape then special marks where it had to be operated on; seeing this I almost droped down and felt faintish, as untill then I had always had a hope it would not be so serious an operation although I had a fear it would.^22^

William Watson Cheyne (whom Mathewson knew because he also was from Shetland) was so concerned about her that he escorted her downstairs and asked the nurse to put her to bed and stay with her a little. Howie and Black assume that Lister's instruction to Mathewson to turn her back on the students and thus look at the same diagram he had drawn for their information was an unintended misstep. They quote John Rudd Leeson, who insists that ‘every stage of the investigation was conducted with the utmost regard for the patient's feelings: technical terms only were used, and nothing was said or suggested that could in any way cause them anxiety or alarm.’^23^

However, in a much later conversation with ‘Nurse Skene’ while Mathewson was in the Convalescent Home in Corstorphine where patients were sent to recover in what was then clean country air, Mathewson is told that Lister's lectures were once ‘all in Latin’. Nurse Skene, who when she began nursing says she was one of Lister's Probationers in Glasgow, got ‘many a good instructive lesson’ from him, and comments that Lister had thought fit to lecture in English ‘so as to diffuse usefull knowledge among the poorer classes which lives far from any Dr & thus would know how to treat their own cases and others. I think it was a shame to change the style of lectures don't you?’^24^ To which Mathewson replies:

No nurse I can't accede to that, as I think it is the best one of changes that Prof Lister has made … . What must have become of many poor sufferers when a press of patients … had to be dismissed ‘cured’ or ‘not’ and not have a ‘Convalascent’ as at present to repair to for 3 or 4 weeks and had to go home … & neither they nor their friends knowing what to do, or how to treat a sore. What a number of deaths must have been then which is delayed & averted now through that very kind considerate act of Prof Lister's. Well Maggie I must say you are right as I can bear witness to that myself, and I believe your eyes & ears has not been in your pocket Maggie, and now I will put you through an Exercise on dressing.^25^

And with that, Nurse Skene proceeds to teach Mathewson some ‘useful knowledge’ of the medical kind. She explains how to make a ‘compress’ from anything—even a handful of dirt—to prevent death from haemorrhage.

In a more detailed and most impressive example of her instruction by staff, Dr. Roxburgh says ‘now Margt I must hear what you understand about dressing your own case?’^26^ Mathewson responds with the following account of the doctor's careful examination of what she has learned:

Well Sir, first I would wash it in the ‘carbolic’, then put a small bit of protextive so big over the wound then the dressing and bandages taking care to bandage from me and when touching an open sore not to touch it with my forefingers. Can you make a dressing. ‘Yes Sir’. Why bandage from you? Because it goes closer, & smoother on, & keeps longer in its position Sir. Yes quite so. Then why not touch a sore with your fore fingers? Because Sir theres poison in the forefingers & thumbs … Yes very good. Now would you ever put wadding next a sore? Its nice & soft isn't it? Yes sir its nice & soft, but it should *never* be put to a sore as theres insects in it (tho' unperceivable to the naked eye) and those insects would cause death if they got into a wound. Yes you are quite right, and had I known sooner you understood those things you should have been away ere now.^27^

In the latest version of the ‘Sketch’, Mathewson omits most of the detail she gives here about the particulars of Listerian wound dressing, writing only that ‘Dr. Roxburgh came & dressed me for the last time and inquired what I understood of dressing my arm. “I am quite satisfied to hear your explanation and thus there's no fear of letting you away now”.’^28^ Even this abbreviated account makes it clear that doctors felt patients must understand how to dress their own wounds, and also *why* certain procedures were followed. In the earlier account, Mathewson's use of the term ‘insects’ for what Lister might have called ‘microbes’, or perhaps ‘vibrios’, demonstrates her ignorance of the terminology currently in use by Listerians in this time of emerging germ theory, but it equally demonstrates her understanding of what we would call the threat of bacterial infection and basic methods of prevention in wound dressing.^29^

Just why Mathewson chose to omit the (to us) fascinating detail about her education in the prevention of wound infection, or her earlier discussion with Nurse Skene, in the later version of her ‘Sketch’ is a matter for speculation. Did she or her friend and copyist, Laurence Williamson, fear this account would make her look ignorant or perhaps even vulgar? Had she learned, perhaps, that what Nurse Skene told her about Lister always lecturing in Latin until he decided to use English for the benefit of the patient's instruction was wrong? Jennifer J. Connor has noted that Lister ‘often lectured in German or French (and sometimes in Latin) for the benefit of his foreign students and visitors’, but believes it unlikely he lectured in Latin in the early years at Glasgow.^30^

Mathewson also omits from the later ‘Sketch’ her account of Nurse Skene's invitation that she return to the Infirmary and enter training to become a nurse herself. Commenting that Mathewson is obviously ‘anxious to learn’, Nurse Skene promises to recommend her to Miss Pringle (one of the ‘Nightingales’ sent from St Thomas' Hospital in London), but Mathewson replies that she fears her arm won't allow that for years to come.^31^ Her inclusion of the invitation, however, implies her pride in having been considered as a potential member of the newly respected profession of nursing.

## ‘I have suffered too long for your pleasure’

After her surgery, performed on 23 March 1877, Mathewson describes her encounter with the ‘cruel dresser’ that demonstrates another aspect of what Lister taught his patients that overturns Foucault's and Jewson's assertions of the silencing of hospital patients. Mathewson's ‘Sketch’ tells us that patients at the Royal Infirmary of Edinburgh were expected to report a staff member or student whose treatment they felt was in any way harmful or abusive, and that patients may even have believed they had the power to have the individual dismissed.^32^

Lister himself did the first dressing change after the surgery, and as he put Mathewson's arm through the full range of motions right then, she experienced such pain as to make her fear she might lose the arm after all:

The pain was undescribable as I had never before felt such pain and I almost fainted from it, & the sweat ran down over me like water, and I felt the arm quite loose from my body, & I felt so weak from the thought of having lost my arm after all!!!^33^

But this was the first dressing change right after the surgery, and she apparently felt that, at least if caused by Lister, such pain simply had to be borne. After Lister left for London in May, Mathewson was at first placed in the care of Dr Roxburgh, whom she describes as being ‘very kind to me’.^34^ But then there was a change of dressers, as every six months students were rotated for duty in the surgical or medical wards. Mathewson was assigned to one of the new student dressers. ‘Until then’, she wrote, ‘I had not known … what a “cruel dresser” meant’:^35^

The first dressing Mr —— made I really thought he had overturned all the ligaments etc. which had then begun to go together. the pain was dreadful and the draw sheet & pillows etc had to be changed for the blood from the wound then the bandages was tight. Miss Logan came in and I was leaning on the table & crying from the pain & soreness. Dear-o-me have you got bad news. No Miss Logan, not in the way you mean, but I have got a cruel dresser!!^36^

She slept but little for the following two nights, and this was the case every time Mr —— (she never names him in any version of the ‘Sketch’) changed her dressings for the next three months. A letter to Mathewson's father dated 11 June 1877 demonstrates that, if anything, her description of the cruelty of this dresser is understated in the ‘Sketch’:

… I mean to ask … if they will let me go to the convalescent now, as then I would (I hope) get free of the fearful Squeezing Mr Hart gives me arm It couldn't be worse any way I think if it should'nt be much better. On Saturday he dressed me sitting on a chair (as I was up before he came, just to see if it would be any better being out of the bed) & it was worse than ever but I tried not to cry out much, he put his knee on my side below my arm and pulled up my arm with both hands the blood ran down over my clothes (thro the places where the tubes was in) it was very sore and painful all Saturday afternoon & night & I hardly sleep't any & it was still sore Yesterday morning but got a little better after that so as I sleept very well last night.^37^

At last, this dresser went on holiday to ‘Vienna’, and while he was away, Roxburgh again took over the dressing changes, for which she was ‘thankful’.^38^ But when Mr Hart returned from his holidays, the torture began again. She told herself that he was ‘trying experiments’ on her case and didn't really have a ‘cruel design’.^39^ But then one day he asked her if she was not ‘wearying to get away’, and she replied:

I am indeed. But your style of dressing is preventing my progress and prolonging my stay here. Well you know yours is a rare case and that's my chance for lessons', Well Sir Indeed, if you presume to dress me any longer so cruel, I am determined to inform on you, as I have that privilege if I choose, thus I am reminding you of that, so as to prepare you for your dismissal, Sir Do you really mean it Margt? I really mean what I say sir, as I have suffered too long for your pleasure & rather than to cause any gentleman lose so important a situation as you are preparing to fill. Well I am much obliged to you for this notice as I know you have it in your power to cause my dismissal, & I beg your pardon, & I shall not be so hard again If you don't inform this time yet.^40^

Despite this bold accusation, there was no difference in the way the student did her dressings thereafter, and when Lister returned from London to visit his patients in Edinburgh again, he was shocked by the condition of Mathewson's wound:

He came and began to undo the bandages on my arm when he came to the sore he stoped & asked whats been doing here (?) Who is the dresser? Mr —— Sir said Dr Rgh Well Mr —— you have not failed to move the joint here (Mr ——'s face got red) and have reopened what was set together Sir which Im sorry for as I expected to see its great progression at this date. Then the pain it must have given the patient!^41^

Lister's reputation for rebuking students severely if he thought they had mistreated a patient is well known. M. Anne Crowther and Marguerite W. Dupree comment, ‘His pained and public reproaches if dressers appeared at all careless or treated patients without proper consideration affected his supporters for the rest of their careers.’^42^ Here Mathewson goes on to report that Lister asks Roxburgh,

Dr did she never report Mr —— to you? ‘Never to me Sir then said to me Did you always feel pain after the dressing? Yes Sir And did you always sleep well the following nights No Sir, I seldom slep't any the following two or three nights Sir.^43^

And Lister then asks her whether she thinks Mr —— did it from cruelty, and she gives her reply, saying that no, she thinks he was just trying to prevent a ‘stiff joint’.

Lister's question as to whether Mathewson had ever reported Mr —— suggests that he expected patients to report mistreatment. Even more significantly, this account suggests that Lister believes that the student might have been deliberately sadistic, manipulating her arm as he did from sheer cruelty. The student may have assumed that because Mathewson was a charity patient, receiving treatment for no charge, he had a right to treat her as a sort of experimental animal, without regard for her pain or progress so long as this would advance ‘science’. Certainly both medical students and practitioners have been accused of sadism throughout medical history, but that charity patients would be encouraged to report a ‘cruel’ dresser and—even more extreme—that the patient might believe she had power to have the dresser dismissed is a dramatic revision of current assessments of patient power in nineteenth-century hospitals as practically nonexistent.^44^

However, Mathewson's version of the ‘cruel dresser’ story is significantly different in the manuscript dated 29 September 1879. She describes both her new dresser's treatment and her response to it in much more succinct and less graphic terms, does not report confronting the student or threatening him with dismissal, and concludes, ‘I felt sure I could not progress under his treatment, and consequently would have to stay a long time still in the Infirmary.’^45^

When Lister returns from London and asks her how she is getting on now, she replies, ‘Thank you Sir, but ordinary.’ When he responds, ‘How is that? You ought to be getting on well by this time’, she comments, ‘I did not answer Professor's question, as I did not wish to inform on Mr —— as there were a great amount of events might come out of it’.^46^ When the Professor undoes the bandage, however, he is described as rebuking Mr H. in even more severe terms than in the earlier ‘Sketch’. And in this version, Mathewson comments, ‘I had to be cautious how I answered Prof. here again, as I believed a great deal would depend on what I said regarding the dressing, as “Many a word in anger spoken, finds its passage back again” says the poet.’^47^

In this later version, then, Mathewson seems to explain her failure to report his cruelty as the fear that the student's resentment at having been publicly rebuked might boomerang on her. Instead of succeeding in having him dismissed, she might simply be subjected to even more cruel treatment thereafter. But a letter written to Dr Cheyne on 4 August 1879, discovered only recently in the Shetland Archives, suggests some alternative interpretations. After explaining how her arm had quite healed up in August 1878, when she had last seen him, she continues with a question about her ‘Sketch’:

Perhaps Sir you have heard ere now that I have written ‘A Sketch of my Eight Months in the Royal Infirmary’ as a cousin of mine got a loan of it and went off to Fetlar, & read it to too many there. Thus you probably have heard of it. It was written in Dec. '78 only not at all in ‘The Infirmary’ as I had nothing noted down there of the many strange things I saw & heard (only ‘The Convalascent’ Meals) all the rest of it is entirely from memory & to be as a help to memory in after years D.V. but of late I have been prevailed on to enquire about having it printed but I have not heard the printers terms as yet only I believe it will be too high for me. It is not written with a view to censure any person in connection with ‘The Infirmary’ as all deserves my highest approbation (with one exception viz Mr —— my ‘dresser’ for 3 months after you left there Sir. But that was experiments he tried I believe and that was the cause of my long stay & which also retarded my progress.)

I had occasion to use your name Sir, very much in my first 3 months there in writing ‘The Sketch’, please Sir have you any objections therefore to its printing if the terms is moderate? I might have come over to Fetlar, & I would have taken it to give you a look through (and a laugh) but I cannot get as one of my brothers is very bad at present from ‘pulmonary Consn.’ Hoping you are well & wishing you every success. I am Dear Sir, Your humble patient, Margaret C. Mathewson^48^

In this letter Mathewson discloses her fear that Cheyne might object to what she has written about *him* in her ‘Sketch’. But she also writes without such hesitation that Mr —— the dresser is the one person connected with the Infirmary who does not deserve her ‘highest approbation’. Who was Mr ——? He was almost certainly David Berry Hart, included in the photograph of Lister's clerks and dressers in 1875 that is reproduced in Crowther and Dupree's *Medical lives in an age of surgical revolution*.^49^ He graduated from the Edinburgh medical school in 1877, is listed as a resident at the Edinburgh Royal Infirmary from November 1878 to May 1879, and went on to become a much respected obstetrician and gynaecologist.^50^

No reply from Cheyne to Mathewson has yet surfaced, but it seems not unlikely that he might either have written or spoken to her concerning the wisdom of publishing a portrait of a recognizable doctor, by then on his way to a respectable career, as not only deliberately causing a patient unnecessary pain but as having humbled himself to her by acknowledging her power to have him dismissed. It is also possible, of course, that patients did not have quite that much power, even though it seems likely that Lister did encourage ward patients to complain if they felt they were being unfairly treated or even abused by medical staff. However, it also seems possible that what Mathewson wrote in the earlier version of her ‘Sketch’, dated 8 August 1879 and so perhaps completed before she had received any response from Cheyne, might have been quite true. It would follow, if that was Lister's policy at Edinburgh, that she would at first have had no compunctions about describing just how little approbation she felt Mr Hart deserved, in comparison with everyone else at the Infirmary. Mathewson's confusing statement in the later version of her ‘Sketch’ about not wanting to inform on Mr —— because ‘there was a great amount of events might come out of it’, sounds as if this might have been hindsight after receiving some cautionary advice from Cheyne, and that it was because of such cautionary advice that she proceeded to revise the manuscript over the next six weeks. Perhaps he did even ‘take a look through (and a laugh)’ at her manuscript and suggested some editing.

## ‘And what a successful case it came to be’

But this is not the end of Mathewson's account of what she had learned as a patient during her time in the Royal Infirmary of Edinburgh. During the summer of 1878 she heard that Cheyne was also at home on the island of Fetlar at that time for his holiday, so she went to see him. It was in her view, I believe, a triumphal visit. In her own words:

In the summer of 78 Dr. Cheyne of Fetlar (who went to London with Prof. Lister) came home. … I went to Fetlar to see him for advice on my arm also to let him see its progress. He probed it to see if it was sound at the bone. I felt it in the shoulder cup, and for some days after it was very sore. He asked if it had ever gathered Yes Sir it gathered three times after I came home.' ‘What did you do?’ I wrote to Dr Chiene Edinburgh and he sent me a drainage tube.' ‘And who put it in?’ ‘Myself, sir, before a glass.’ He was very much amused and surprised at this, then had lots of questions; then said, Well it is quite sound at the bone and it will doubtless get to be as strong as the other yet, and what a successful case it came to be and I am so glad to see it.'^51^

Mathewson added, in this later version of her ‘Sketch’: ‘It healed quite up in August and since feels much stronger. It was 17 months healing. Now I can do any sort of indoor work, even washing clothes, etc. And looking back through this ordeal of trouble, how I am laid to wonder, and adore God's love’, and she concludes with a quotation from a hymn.^52^ Little more than a year later, however, her father wrote to his remaining children, Joanna, Laurence and others, ‘I write to you at present to let you know that I followed my Dear Margaret your Sister to the Grave in the Asaylum [sic] in MidYell on the evening of Saturday the 2^nd^ October’.^53^ He lost three children in that single year of 1880: Arthur died at age 41 on 20 February 1880, Margaret at age 32 on 28 September 1880, and Walter at age 38 on 31 October 1880; the two brothers most probably, like Margaret, succumbed to tuberculosis.^54^

## Conclusion

Margaret Mathewson's ‘Sketch’ was not published, even in excerpt form, for more than 100 years, and the excerpts that were published came only from a manuscript copy that, as I have explained, is a revision of earlier versions. In those earlier versions this patient documents an aspect of Lister and his staff's treatment of his patients that I believe has not been described anywhere else. She tells us that staff members were expected to teach patients the medical knowledge and nursing skills they needed to know to care for themselves and also to help their family and friends in remote locations in Scotland where trained medical practitioners were often unavailable. On the basis of Mathewson's account, Lister seems to have been practising a form of public health medicine.

However, still more radically, in Mathewson's account Lister gives his patients complaint power—a kind of patients' rights whose very notion has been thought to be a far more recent development. Was Lister unique in encouraging patients to complain about abusive treatment? Or is this an aspect of nineteenth-century hospital policy that was more widespread in British hospitals than has been suspected? Margaret Mathewson's ‘Sketch’ re-frames our image of Victorian hospital patients and should inspire further investigation of the history of patient power in British hospitals. And although Lister's fame as a teacher of medical students is already well known, and as the articles in this collection by Ruth Richardson, Michael Warboys and Thomas Schlich demonstrate his equally strong interest in teaching medical colleagues by performance and by precise, detailed description,^55^ so Mathewson's ‘Sketch’ shows us that he expanded his teaching to include that lowliest group of students in the clinic, the ward patients.

## Notes

1 It has not been possible to determine the precise date of this visit by Lister to the Infirmary, other than to state that it was some time in June—probably after his formal appointment as the new Chair of Clinical Surgery on 18 June—or July before 26 July, when Mathewson was moved to the Convalescent Home in Corstorphine.

2 Margaret Mathewson, ‘Sketch of Eight Months a patient, in the Royal Infirmary of Edinburgh, A.D. 1877’, dated 8 August 1879, holograph manuscript photocopy held in Shetland Archives, SA2/340, p. 111.

3 Mathewson's residence in the Convalescent Home also coincided with Lister's visit to Corstorphine in August 1877 to collect milk from a dairy cow to use for his studies on *Bacterium lactis*, so brilliantly presented at his Introductory Address, although she probably knew nothing of that either. (See Ruth Richardson, ‘Inflammation, suppuration, putrefaction, fermentation: Joseph Lister's microbiology’, *Notes Rec. R. Soc.* **67** (this issue) (http://dx.doi.org/rsnr.2013.0034).)

4 This earliest version of Mathewson's narrative, dated 15 December 1878 (D1/557/5), and another version dated 26 July 1879 and with the slightly different title of ‘A Help to Memory, or a Sketch of Eight Months a Patient in the Royal Infirmary of Edinburgh’ (D1/557/4), have very recently (in March 2013) been discovered and deposited in the Shetland Archives.

5 Trisha Greenhalgh and Brian Hurwitz, ‘Why study narrative?’, in *Narrative based medicine: dialogue and discourse in clinical practice* (ed. Trisha Greenhalgh and Brian Hurwitz), pp. 3–16 (BMJ Books, London, 1998), at pp. 4–5.

6 Mathewson, *op. cit.* (note 4).

7 W. B. Howie and S. A. B. Black, ‘Sidelights on Lister: a patient's account of Lister's care’, *J. Hist. Med. Allied Sci.* **32**, 239–251 (1977). Howie and Black also published a very brief version of this article, ‘Hospital life a century ago’, *Br. Med. J.* **ii** 515–517 (1976).

8 Sir Rickman John Godlee, *Lord Lister* (Clarendon Press, Oxford, 1917); John Rudd Leeson, *Lister as I knew him* (William Wood & Co., New York, 1927).

9 Richard B. Fisher, *Joseph Lister 1827–1912* (MacDonald & Jane's, London, 1977).

10 Martin Goldman, *Lister Ward* (Adam Hilger, Bristol, 1987).

11 Margaret Mathewson, ‘A Sketch of Eight Months a Patient in the Royal Infirmary of Edinburgh, A.D. 1877’, dated 27 September 1879, first six pages holograph, the remainder copied by Laurence Williamson, Shetland Archives, D.7/77.

12 Mathewson, *op. cit.* (note 2), n.p.

13 Michel Foucault, *The birth of the clinic: an archaeology of medical knowledge* (tr. A. M. Sheridan Smith) (Vintage Books, New York, 1994; orig. pub. Pantheon Books, 1973; orig. French pub. Presses Universitaires de France, 1963); Nicholas D. Jewson, ‘The disappearance of the sick-man from medical cosmology, 1770–1870’, *Sociology* **10**, 225–244 (1976). See also David Armstrong, ‘The patient's view’, *Social Sci. Med.* **18**, 737–744 (1984); Roy Porter, ‘The patient's view: doing medical history from below’, *Theory and Society* **14**, 175–198 (1985); Mary A. Fissell, ‘The patient's perspective’, ch. 8 in *Patients, power, and the poor in eighteenth-century Bristol* (Cambridge University Press, 1991), pp. 148–170.

14 Uncatalogued document (untitled); Shetland Archives.

15 However, Guenter B. Risse, in his history of hospitals that is focused on patient histories, does comment that Nurse Skeene, who had been a probationer under Lister, ‘insisted on educating her patients in dressing their own wounds and dealing with elementary first aid’, because his source is a photocopy held at Lothian Archives (in which Edinburgh Royal Infirmary archives are now included). This photocopy is a copy of the photocopy of Mathewson's manuscript dated 7 August 1879 held at the Shetland Archives. Risse does not refer to the difference between this version of the ‘Sketch’ and the later one used by Goldman in *Mending bodies, saving souls: a history of hospitals* (Oxford University Press, 1999, p. 386).

16 Uncatalogued letter dated 6 March 1877, Edinburgh Royal Infirmary, to ‘Dear Father, Brother & Sister’, signed ‘Margaret C. Mathewson’. Shetland Archives.

17 Mathewson, *op. cit.* (note 2), pp. 37–38.

18 *Ibid.*, p. 69.

19 *Ibid.*, p. 41.

20 *Ibid.*, p. 41.

21 *Ibid.*, p. 41.

22 *Ibid.*, p. 42.

23 Howie and Black, *op. cit.* (note 7), p. 244.

24 Mathewson, *op. cit.* (note 2), pp. 169–170.

25 *Ibid.*, pp. 170–171.

26 *Ibid.*, p. 176.

27 *Ibid.*, pp. 176–177.

28 Mathewson, *op. cit.* (note 11), p. 87.

29 Worboys indicates that in the early 1870s Lister pictured germs as very simple and mutable organisms whose development changed according to their environment: Michael Worboys, *Spreading germs: disease theories and medical practice in Britain, 1865–1900* (Cambridge University Press, 2000), pp. 96–97. Santer, however, points out that Lister's paper ‘On the lactic fermentation and its bearing on pathology’, published in *Transactions of the Pathological Society of London* for the session 1877–78, ‘demonstrated that a specific organism caused a specific phenomenon’: Melvin Santer, ‘Joseph Lister: first use of a bacterium as a “model organism” to illustrate the cause of infectious disease of humans’, *Notes Rec. R. Society* **64**, 59–65 (2010), at p. 63. Lister was working with a pure culture of *Bacterium lactis*. This suggests a significant advance in Lister's understanding of microorganisms that happened to coincide with the period of Mathewson's hospitalization, and might have been discussed with his hospital staff. See also Richardson, *op. cit.* (note 3).

30 Jennifer J. Connor, ‘Being Lister: ethos and Victorian medical discourse’, *Med. Humanities* **34**, 3–10 (2008), at p. 4; personal communication, 16 March 2012. However, Bonner notes that Robert J. Graves, in ‘On Clinical Instruction, with a Comparative Estimate of the Mode in which it is conducted in the British and Continental Schools’, *Lond. Med. Gaz.* **10**, 404–406 (1832), states that professor and students met in a classroom around a large table, where patients were introduced separately from ‘an antechamber’. Once seated, the professor would call on a pupil to examine the patient, then to declare aloud, in the Latin tongue, the diagnosis, prognosis, and ‘methodus curandi’, whereupon the professor would interrogate both patient and pupil and explain the disease'. Thomas Neville Bonner, *Becoming a physician: medical education in Britain, France, Germany, and the United States, 1750–1945* (Oxford University Press, 1995), p. 115, note 43.

31 Mathewson, *op. cit.* (note 2), p. 171.

32 Howie states that ‘from the beginning of the hospital movement it was appreciated that there was a need for a system through which complaints could be made and wrongs redressed.’ At the Salop Infirmary in Shrewsbury, the rules for patients even began with a section headed ‘What the Patient May Expect’, and included such items as ‘the greatest civility and attention from the house surgeon, the matron, the nurses, and every other person employed by the charity, together with regular attendance from the physicians, the surgeons, and the house surgeon.’ But in fact the greatest number of complaints was brought against patients, who could be discharged for bad behaviour. Although a few complaints were brought by patients against senior members of the medical staff, Howie comments that it was surprising that any were made as, if the complaint was found to be false, the patient might be denied future admission to the hospital on any grounds whatever. W. B. Howie, ‘Complaints and complaint procedures in the eighteenth- and early nineteenth-century provincial hospitals in England’, *Med. Hist.* **25**, 345–362 (1981), at pp. 345, 346 and 356. Risse comments, ‘lack of proper complaint procedures also hampered the airing of grievances, since patients almost never dared to utter criticisms because of fear that the staff would retaliate’: Guenter B. Risse, *Hospital life in Enlightenment Scotland: care and teaching at the Royal Infirmary of Edinburgh* (Cambridge University Press, 1986), p. 24.

33 Mathewson, *op. cit.* (note 2), p. 74.

34 *Ibid.*, p. 107.

35 *Ibid.*, p. 107.

36 *Ibid.*, pp. 107–108.

37 Shetland Archives, D23/151/43/1-45.

38 Mathewson, *op. cit.* (note 2), p. 109.

39 *Ibid.*, p. 109.

40 *Ibid.*, pp. 109–110.

41 *Ibid.*, p. 110.

42 M. Anne Crowther and Marguerite W. Dupree, *Medical lives in the age of surgical revolution* (Cambridge University Press, 2007), p. 102.

43 Mathewson, *op. cit.* (note 2), pp. 110–111.

44 Wilde points out that ‘there is now a very considerable body of work that emphasizes the varied and negotiated nature of nineteenth- and early-twentieth-century clinical relationships and the importance of patients as autonomous actors’, but her article deals with private, paying patients, who began to enter hospitals voluntarily in the late nineteenth century. In addition, although she argues that patients had more authority in terms of giving or not giving consent to surgery, she does not consider whether patients, especially non-paying or charity patients, might have had the authority to report abuse by medical staff and cause their dismissal. Sally Wilde, ‘Truth, trust, and confidence in surgery, 1890–1910: patient autonomy, communication, and consent’, *Bull. Hist. Med.* **83**, 302–330 (2009), at p. 307.

45 Mathewson, *op. cit.* (note 11), pp. 62–63.

46 *Ibid.*, p. 63.

47 *Ibid.*, pp. 63–64.

48 Uncatalogued letter to Dr Cheyne on Fetlar from Margaret Mathewson, dated 4 August 1879.

49 Crowther and Dupree, *op. cit.* (note 42), p. 4.

50 I thank the Archivist, Laura Gould, at Lothian Health Services Archive in Edinburgh, as well as Anne Crowther and Marguerite Dupree, for directing my attention to this information.

51 Mathewson, *op. cit.* (note 11), p. 92.

52 *Ibid.*, p. 93.

53 Shetland Archives, D1/411/3/6. Letter dated 18 October 1880.

54 Goldman, *op. cit.* (note 10), pp. 144–145.

55 Richardson, *op. cit.* (note 3); Michael Worboys, ‘Joseph Lister and the performance of antiseptic surgery’, *Notes Rec. R. Soc.* **67** (this issue) (http://dx.doi.org/rsnr.2013.0028); Thomas Schlich, ‘Farmer to industrialist: Lister's antisepsis and the making of modern surgery in Germany’, *Notes Rec. R. Soc.* **67** (this issue) (http://dx.doi.org/rsnr.2013.0032).

